# Measuring scientific coherence between global neglected tropical disease research and population health indicators: a 25-year meta-research study

**DOI:** 10.3389/frma.2026.1766718

**Published:** 2026-06-18

**Authors:** David A. Hernandez-Paez, Kevin Fernando Montoya-Quintero, Jhon Víctor Vidal-Durango, Ivan David Lozada-Martínez

**Affiliations:** 1 Center for Meta-Research and Scientometrics in Biomedical Sciences, Barranquilla, Colombia; 2 Facultad de Ciencias para la Salud, Universidad de Manizales, Manizales, Colombia; 3 Facultad de Ciencias Básicas, Universidad de Córdoba, Montería, Colombia; 4 Biomedical Scientometrics and Evidence-Based Research Unit, Department of Health Sciences, Universidad de la Costa, Barranquilla, Colombia; 5 Clínica Iberoamérica, Clínica Colsanitas S.A., Barranquilla, Colombia

**Keywords:** clinical epidemiology, global health, health status indicators, meta-research, neglected tropical diseases

## Abstract

**Introduction:**

Despite major investments in neglected tropical diseases (NTDs) research over the past two decades, it remains unclear whether global scientific production aligns with population-level health indicators. To address this gap, we designed and implemented a comprehensive meta-research analysis pipeline to quantify the scientific coherence between global NTDs research activity and epidemiological indicators across World Health Organization (WHO) regions from 2000–2024.

**Methods:**

Using this pipeline, we linked 1,07,251 NTDrelated publications to 23 disease-specific indicators from WHO, World Bank, and Our World in Data databases. Region-year panels were analyzed using bivariate regressions and hierarchical mixed-eects models, with publication count as the predictor and disease indicators as dependent variables, adjusting for temporal trends and regional clustering.

**Results:**

Higher research activity was significantly associated with reductions in HIV incidence, tuberculosis incidence, malaria incidence, and schistosomiasis treatment requirements, with hierarchical models confirming consistent protective associations and moderate heterogeneity across regions. In contrast, indicators such as antibiotic consumption and total DALYs showed incoherent or null relationships, suggesting persistent misalignment between research intensity and broader health indicators.

**Discussion:**

By introducing a reproducible, data-driven framework for assessing research-indicators coherence, this study shows that although NTDs research contributes to epidemiological improvements, coherence remains incomplete. Incorporating coherence metrics into global health assessment may help align scientific investment with public health needs and enhance the translational impact of research systems.

## 1 Introduction

For scientific research has become a cornerstone of modern health systems, guiding clinical and policy decisions through the continuous generation of evidence (Mahara et al., 2023). Yet, whether scientific production aligns with tangible improvements in population health remains an open question (Penfield et al., 2014), particularly in areas where diseases persist despite decades of research investment (Penfield et al., 2014). This gap between knowledge generation and real-world outcomes lies at the center of contemporary meta-research (Evans et al., 2014), which seeks to examine how, and how well, science fulfills its purpose of improving human health (Evans et al., 2014).

Neglected tropical diseases (NTDs) represent a paradigmatic case of this dilemma (World Health Organization, 2024). Affecting more than one billion people worldwide (World Health Organization, 2024), NTDs are both a biomedical and epistemic challenge: they cluster where poverty, weak health systems, and limited research capacity intersect (World Health Organization, 2024). Despite their inclusion in global health agendas and the growing number of publications dedicated to them (Tebano et al., 2024), many NTDs continue to cause substantial morbidity and mortality (World Health Organization, 2024). This paradox raises a critical question: to what extent scientific research on NTDs evolves coherently with population-level health indicators in affected populations?

Previous studies have evaluated the evolution of NTDs research from a bibliometric perspective (Fontecha et al., 2021; Tebano et al., 2024), describing publication trends, geographical disparities, and collaboration patterns (Fontecha et al., 2021; Tebano et al., 2024). However, such analyses rarely address the functional coherence between research efforts and epidemiological realities. In other words, they assess what is being published (Fontecha et al., 2021; Tebano et al., 2024), but not whether research activity is associated with measurable improvements in disease burden or access to care. From a meta-research perspective, this coherence, or lack thereof, constitutes a key indicator of the efficiency and equity of the global knowledge system (Thonon et al., 2015).

The discipline of clinical epidemiology offers a suitable lens for such an inquiry (Hoshino, 2020). By integrating methodological rigor with a population perspective, it allows researchers to examine how evidence is produced, validated, and translated into health outcomes (Kirby et al., 2025; Revolledo Caicedo et al., 2025; Romero-Freile et al., 2025). Within this framework, research activity itself can be conceptualized as a health system variable, a measurable component of global responses to disease (Lozada-Martinez et al., 2025b). Assessing its relationship with epidemiological indicators may thus provide insights into whether research systems operate coherently with the needs of affected populations (Kirby et al., 2025; Revolledo Caicedo et al., 2025; Romero-Freile et al., 2025).

Meta-research has emphasized the need for studies that evaluate the performance of the evidence ecosystem: the set of actors, practices, and infrastructures that generate, disseminate, and apply research (Lozada-Martinez et al., 2025b). Yet, empirical models capable of linking scientific output with health indicators remain scarce. This scarcity reflects both conceptual and data challenges: bibliometric indicators are often disconnected from epidemiological surveillance, and global health metrics rarely incorporate research capacity as a predictor variable (Kirby et al., 2025).

This study addresses that gap by proposing a quantitative framework to evaluate the coherence between global scientific production and population health indicators in NTDs. Using more than two decades of data across World Health Organization (WHO) regions, we integrate bibliometric information with epidemiological indicators to test whether greater research activity aligns with improvements in disease-specific indicators such as incidence, mortality, and treatment coverage.

Beyond its specific findings, this work contributes methodologically to the emerging field of meta-epidemiology and meta-research of research systems (Zhang, 2016), the study of how knowledge production behaves as a macro-level correlate of health. By measuring coherence between research and indicators, we aim to examine whether science is not only productive but responsive to and aligned with global health priorities (Kirby et al., 2025). In doing so, this study extends the mission of clinical epidemiology from the appraisal of evidence for individual interventions to the evaluation of the evidence system itself.

Importantly, this study does not aim to establish causal effects between scientific production and population health indicators. Instead, it addresses a well-recognized methodological gap by proposing an ecological and longitudinal proxy of scientific coherence, defined as the degree of alignment between scientific production and a broader set of population-level health, scientific, and societal indicators, rather than being restricted solely to disease burden metrics.

## 2 Methods

### 2.1 Study design and conceptual framework

We conducted a longitudinal meta-research analysis to examine the coherence between global research activity on NTDs and population-level health indicators. This study conceptualizes research coherence as the degree to which scientific production is aligned with improvements in disease burden indicators over time (Kirby et al., 2025). The analysis integrates bibliometric metadata with epidemiological and population indicators from publicly available databases between 2000 and 2024, using the WHO regional classification as the primary analytical unit. The approach aligns with the emerging field of meta-research and meta-epidemiology, focusing on the performance and alignment of research systems in global health (Zhang, 2016).

### 2.2 Data sources

Publication data were obtained from a comprehensive search of the Scopus database, identifying 1,35,129 NTDs-related publications between 1904 and 2024 (Supplementary material 1). However, analytical models were restricted to the period 2000–2024 to ensure consistency and completeness of epidemiological indicators. After excluding records without author affiliation or country information, 1,07,251 publications were retained. Each record included publication year, first-author country, and metadata used to estimate regional publication counts per year.

Country attribution was based on first-author affiliation as a pragmatic and reproducible proxy for large-scale, longitudinal analyses (Gauffriau et al., 2008). Fractional counting approaches are widely used in scientometrics and may better represent shared authorship contributions in collaborative publications (Perianes-Rodriguez et al., 2016). However, implementing fractional counting consistently across multi-decadal, global datasets remains challenging due to variability in affiliation formats, incomplete historical metadata, and the need for harmonized country-level aggregation over long time series.

In this context, first-author affiliation provides a transparent and reproducible approach that has been widely adopted in large-scale bibliometric studies when the objective is to estimate regional research activity rather than individual contribution patterns. The implications of this choice should be considered when interpreting country-level estimates.

Country-level indicators were extracted from the Global Health Observatory (WHO), World Bank Open Data, and Our World in Data databases. A total of 75 indicators spanning eight domains were compiled: (1) health systems and healthcare access; (2) disease burden and infectious diseases; (3) demographic and population health; (4) economy and poverty; (5) education and development; (6) governance and political context; (7) water, sanitation, and hygiene; and (8) research and development.

For this analysis, we focused on 23 indicators related to infectious diseases and general health indicators (e.g., Human Immunodeficiency Virus [HIV] incidence, tuberculosis treatment success rate, malaria incidence, deaths from NTDs, and disability-adjusted life years [DALYs]). This subset was selected based on relevance to communicable disease dynamics, availability of continuous data across the study period (2000–2024), and acceptable levels of missingness, enabling robust longitudinal modeling.

Each indicator was matched by country and publication year, standardized to WHO regions, and merged with the publication dataset. Missing values were not imputed; instead, models were estimated using complete cases only. The full list of variables and data sources is provided in Supplementary Table 1.

### 2.3 Variables and operational definitions

The main predictor was the annual count of NTDs-related publications per WHO region. Dependent variables were epidemiological indicators representing disease burden, morbidity, mortality, or treatment coverage. Covariates included publication year (to adjust for secular trends) and WHO region as a clustering factor.

In this study, publication counts are not intended to measure research quality, topical specificity, or translational effectiveness. Rather, they are used as an ecological proxy to capture the intensity of knowledge production at the system level across regions and over time.

Indicators were automatically classified as predictors using a keyword-based algorithm that distinguished between health results (e.g., prevalence, deaths, and incidence) and system-level resources (e.g., expenditure, physicians, research, and development funding). The classification rules, validation procedures, and diagnostic summaries are detailed in Supplementary material 2.

Although the bibliometric component focused on NTD-related publications, we intentionally considered a broader set of population-level burden indicators to capture scientific coherence at the system level, consistent with a social determinants of health perspective rather than disease-specific impact assessment.

### 2.4 Statistical analysis

Analyses were conducted in three complementary stages.

#### 2.4.1 Region-specific regression models

For each of the six WHO regions (Africa, Americas, Eastern Mediterranean, Europe, South-East Asia, and Western Pacific), analyses were conducted at the country-year level, fitting region-specific models to estimate the association between NTD-related publication output and population-level indicators. Model type was selected algorithmically using pre-specified statistical rules based on the empirical distribution of the dependent variable, including assessment of scale, skewness, and dispersion: (A) Gaussian models for approximately normal results; (B) log-transformed Gaussian models for positively skewed continuous results; (C) Poisson or negative binomial models for equidispersed or overdispersed count data; (D) logistic models for binary results; and (E) Beta regression for proportions bounded in (0,1) (Fernandez and Vatcheva, 2022).

Coefficients were reported as unstandardized β, odds ratios (OR), incidence-rate ratios (IRR), or percent change (100 × [e^β^ – 1]), with corresponding 95% confidence intervals (CI).

To control for multiple testing, *p*-values were adjusted using the Benjamini–Hochberg method (Ferreira, 2007) within each region, with statistical significance defined as *p* <0.05 after correction.

Measures of fit [*R*^2^, pseudo-*R*^2^, Akaike Information Criterion (Portet, 2020)] were retained for interpretability. All regression diagnostics, code routines, and model outputs are presented in Supplementary material 2.

#### 2.4.2 Hierarchical mixed-effects models

To account for regional heterogeneity and temporal dependency, we next fitted mixed-effects models pooling all region-year observations. For each indicator, we estimated models of the form: [dependent variable ~ publication count + year + (1 | region)], using the appropriate distributional family (Gaussian, log-normal, Poisson, negative binomial, or beta). Random intercepts captured unobserved between-region variation.

Coefficients were expressed as β, OR, or IRR with 95% CIs. Model performance was summarized using marginal and conditional *R*^2^ statistics. Where convergence issues occurred, a non-hierarchical analog was fitted.

This stage allowed a global assessment of coherence while adjusting for time and clustering effects. Full results and sensitivity checks appear in Supplementary material 2.

#### 2.4.3 Sensitivity and robustness analyses

We performed several robustness checks to verify the stability of estimates: (1) repeating analyses after excluding indicators with >50% missing data; (2) re-estimating models using alternative predictor definitions (e.g., publication share normalized by total scientific output); and (3) testing the reverse specification, where research output was modeled as the dependent variable to explore potential bidirectional relationships.

Results were consistent across specifications, with minor variation in association magnitude but preserved directionality. All analyses were conducted in *R* (version 4.3) using the lm, glm, MASS, betareg, lme4, and glmmTMB packages. Reproducible scripts and data are publicly available at https://doi.org/10.5281/zenodo.17467257.

#### 2.4.4 Transparency, reproducibility, and ethics

This study used only publicly accessible, aggregated data and did not involve human participants; therefore, ethical review was not required. The analytic protocol was designed according to principles of open and reproducible science. All datasets, scripts, and Supplementary material have been deposited in an open repository to ensure transparency and facilitate independent replication. Additionally, this work followed the STROBE (Strengthening the Reporting of Observational Studies in Epidemiology) guidelines.

## 3 Results

### 3.1 Descriptive overview

**Figure 1:**
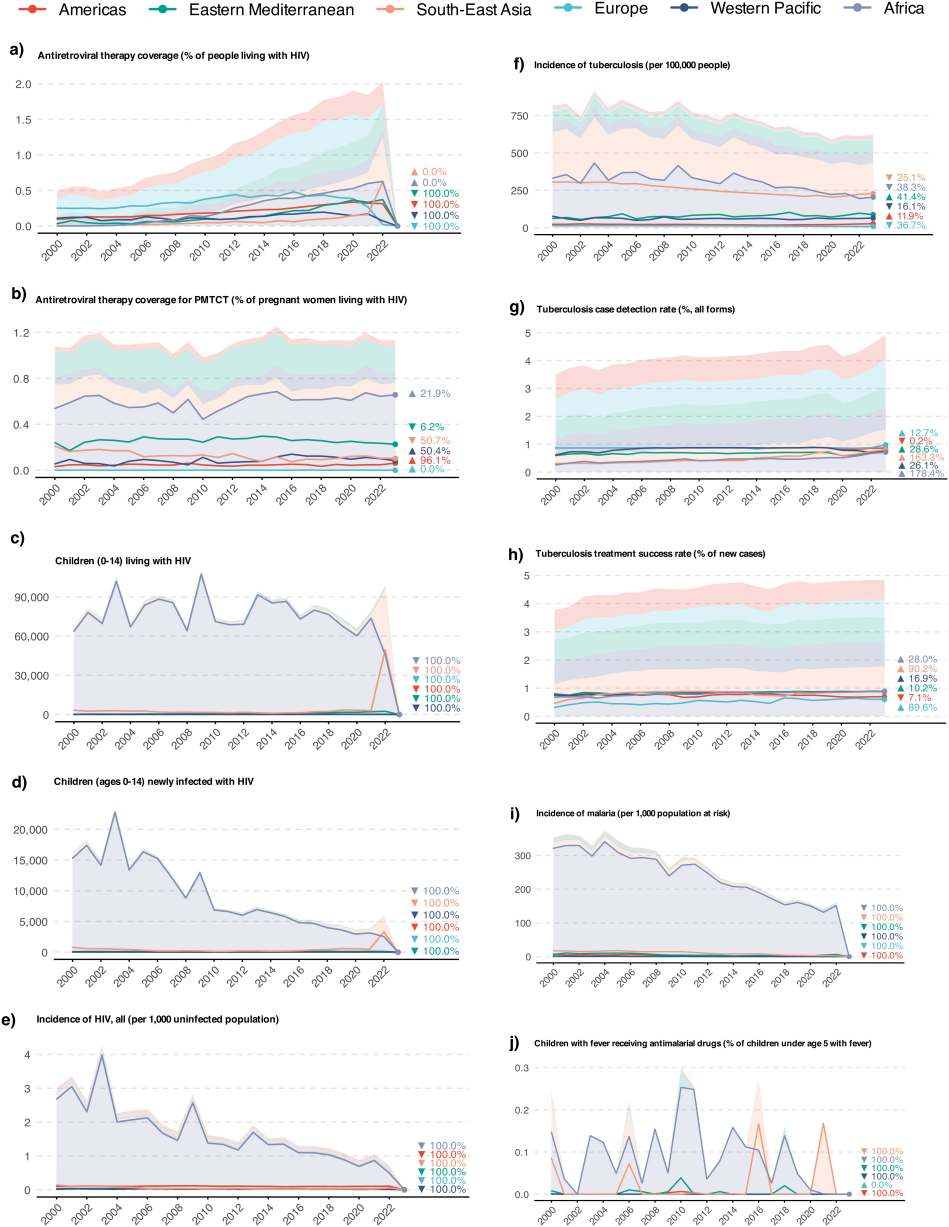
Temporal trends in HIV, tuberculosis and malaria indicators across WHO regions. Time-series plots depicting five HIV-related indicators across the six World Health Organization regions. Solid lines represent regional mean values, while shaded bands denote data ranges. Percentage changes and directional markers (▴ increase, ▾ decrease) at line endpoints indicate the relative difference between the earliest and most recent available data points. **(a)** Antiretroviral therapy coverage (% of people living with HIV); **(b)** Antiretroviral therapy coverage for PMTCT (% of pregnant women living with HIV); **(c)** Children (0–14) living with HIV; **(d)** Children (ages 0–14) newly infected with HIV; **(e)** Incidence of HIV, all (per 1,000 uninfected population); **(f)** Incidence of tuberculosis (per 100,000 people); **(g)** Tuberculosis case detection rate (%, all forms); **(h)** Tuberculosis treatment success rate (% of new cases); **(i)** Incidence of malaria (per 1,000 population at risk); and **(j)** Children with fever receiving antimalarial drugs (% of children under age 5 with fever). DALYs, disability-adjusted life years; NTDs, neglected tropical diseases; PMTCT, prevention of mother-to-child transmission.

**Figure 2:**
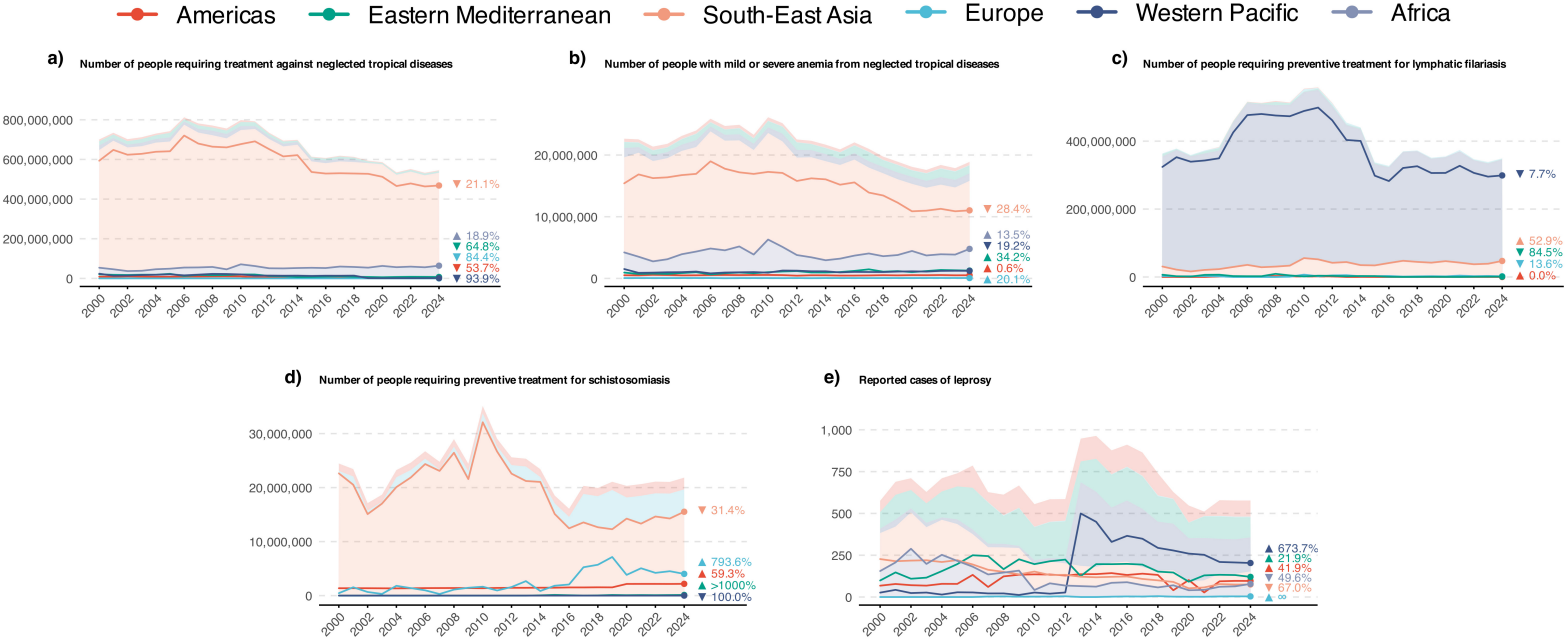
Temporal trends in neglected tropical diseases indicators across WHO regions. Time-series plots depicting five neglected-tropical-diseases-related indicators across the six World Health Organization regions. Solid lines represent regional mean values, while shaded bands denote data ranges. Percentage changes and directional markers (▴ increase, ▾ decrease) at line endpoints indicate the relative difference between the earliest and most recent available data points. **(a)** Number of people requiring treatment against neglected tropical diseases; **(b)** Number of people with mild or severe anemia from neglected tropical diseases; **(c)** Number of people requiring preventive treatment for lymphatic filariasis; **(d)** Number of people requiring preventive treatment for schistosomiasis; and **(e)** Reported cases of leprosy.

**Figure 3:**
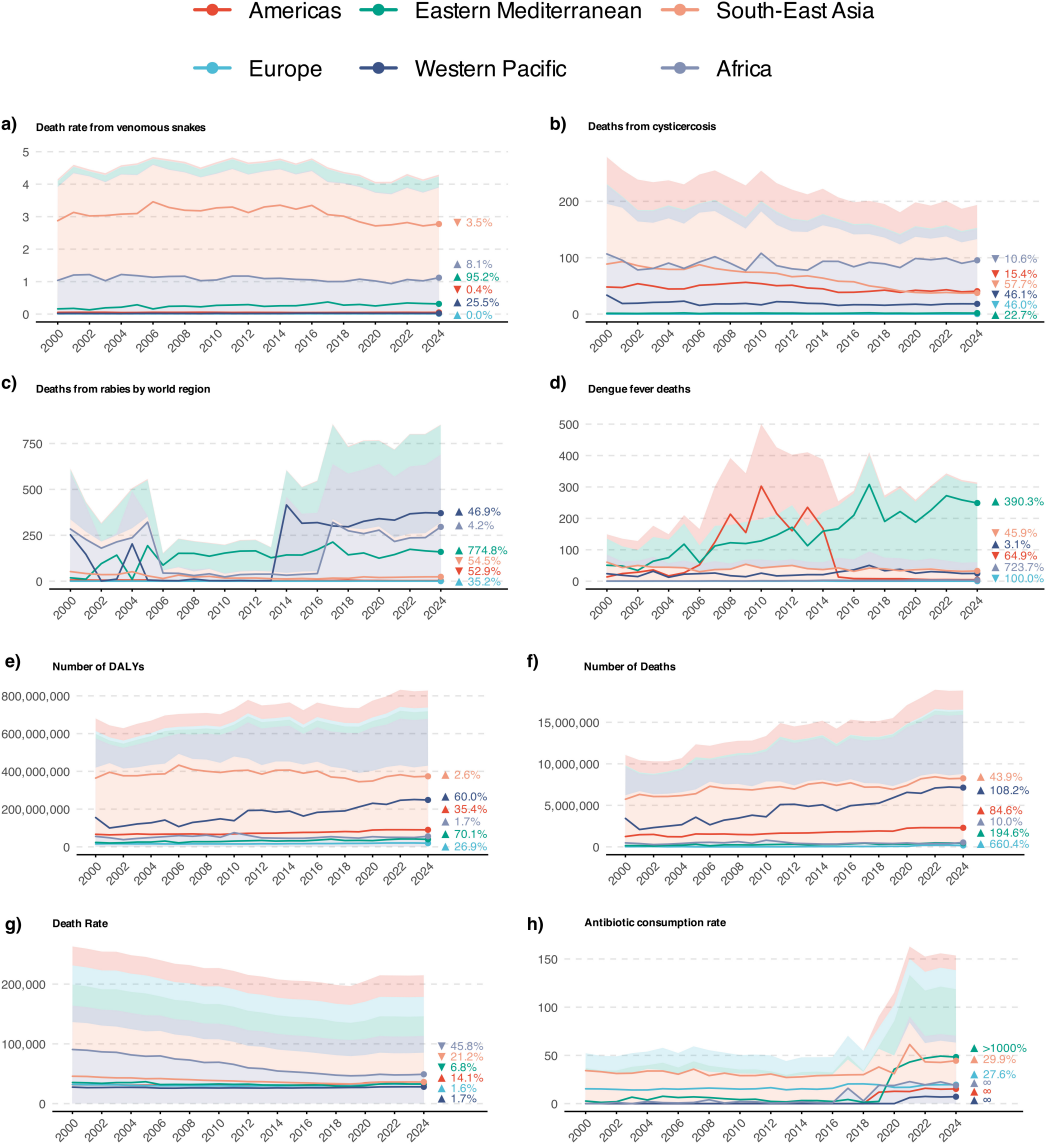
Temporal trends in neglected tropical disease mortality and general health indicators across WHO regions. Time-series plots showing four mortality-related indicators for neglected tropical diseases across the six World Health Organization regions. Solid lines represent regional mean values, while shaded areas denote data ranges. Percentage changes and directional markers (▴ increase, ▾ decrease) at line endpoints indicate the relative difference between the earliest and most recent available data points. **(a)** Death rate from venomous snakes; **(b)** Deaths from cysticercosis; **(c)** Deaths from rabies by world region; **(d)** Dengue fever deaths; **(e)** Number of DALYs; **(f)** Number of Deaths; **(g)** Death Rate; and **(h)** Antibiotic consumption rate. DALYs, disability-adjusted life years.

Between 2000 and 2024, global research on NTDs expanded markedly, with more than 107,000 publications identified after filtering. Output increased consistently across all six WHO regions, most notably in the Americas and Western Pacific, while Africa and South-East Asia exhibited more modest but accelerated growth during the last decade. This expansion coincided with a general decline in major communicable disease indicators, though the magnitude and direction of changes varied substantially by region (Figures 1–3).

Indicators related to HIV, tuberculosis, and malaria showed the most consistent downward trends globally, whereas metrics describing NTDs burden (e.g., population requiring preventive chemotherapy or anemia due to parasitic infection) demonstrated slower and regionally heterogeneous improvement.

### 3.2 Associations between NTDs research and disease-specific indicators

#### 3.2.1 HIV indicators

**Figure 4:**
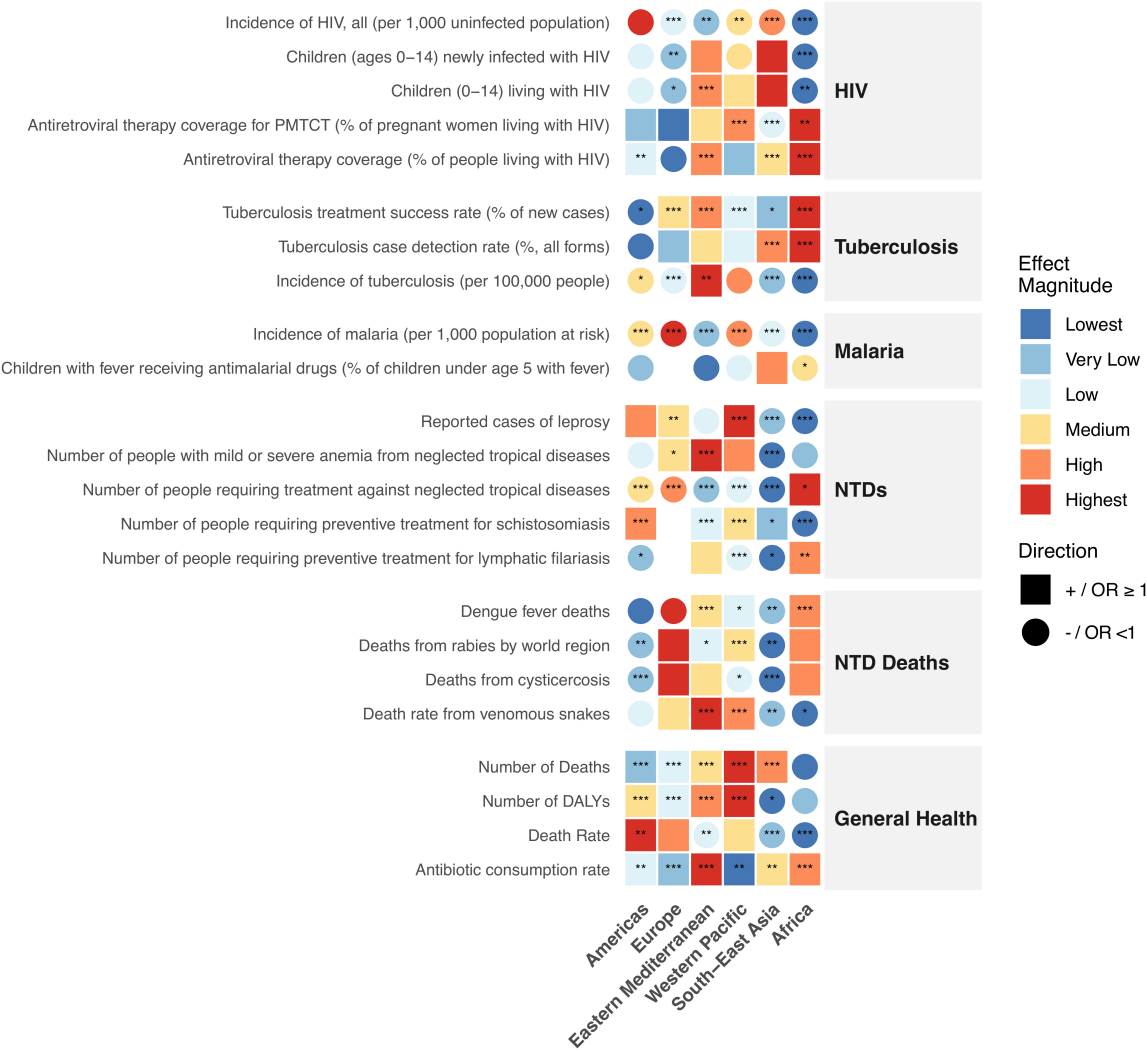
Effect of scientific publications on neglected diseases across health indicators by WHO regions. The heatmap displays regression coefficients from linear models examining the relationship between neglected diseases research output and 23 health indicators categorized into six domains: HIV, tuberculosis, malaria, NTDs (neglected tropical diseases), NTD deaths, and general health. Countries are grouped according to the six World Health Organization regions. Color intensity represents coefficient magnitude (red = highest, dark blue = lowest). Shape indicates effect direction (squares = positive associations, circles = negative associations). Statistical significance is denoted by asterisks (*p <0.05; **p <0.01; ***p <0.001). Blank cells indicate insufficient data for analysis. DALYs, disability-adjusted life years; NTDs, neglected tropical diseases; PMTCT, prevention of mother-to-child transmission.

Across WHO regions, higher NTDs research output was generally associated with improved HIV indicators (Figure 4). In Africa, each additional NTD-related publication corresponded to a 0.011-point decrease in HIV incidence per 1,000 uninfected individuals (β = −0.011; 95% CI: −0.016 to −0.007; *p* <0.001). Similarly, the number of children living with HIV declined by approximately 78 cases per additional publication (β = −78.08; 95% CI: −103.98 to −52.19).

Regarding treatment coverage, NTDs research was positively associated with the odds of antiretroviral therapy (ART) coverage for prevention of mother-to-child transmission in Africa (OR = 1.002; 95% CI: 1.001–1.004; *p* <0.01), but inversely associated in South-East Asia (OR = 0.999; 95% CI: 0.998–0.999).

#### 3.2.2 Tuberculosis indicators

In South-East Asia, each additional NTD-related publication was associated with a reduction of 0.165 cases per 1,00,000 population (β = −0.165; 95% CI: −0.188 to −0.142; *p* <0.001), while in Africa the association was even greater (β = −0.878; 95% CI: −1.122 to −0.635; *p* <0.001). Treatment success improved in Africa (OR = 1.006; 95% CI: 1.004–1.008; *p* <0.001) but declined modestly in the Americas (OR = 0.998; 95% CI: 0.997–1.000).

#### 3.2.3 Malaria indicators

Malaria indicators displayed the strongest overall coherence signal. In all WHO regions, increased NTDs research output was linked to reduced malaria incidence, with the largest association in Africa, where the estimated decline exceeded one case per 1,000 individuals at risk per additional publication. However, no consistent associations emerged for antimalarial treatment coverage among children under five, suggesting that advances in prevention and control may not directly mirror clinical treatment patterns.

#### 3.2.4 NTDs burden indicators

Associations between research activity and direct NTDs burden were mixed. In Africa and South-East Asia, greater research output corresponded to fewer reported leprosy cases and smaller populations requiring preventive treatment for schistosomiasis, indicating partial coherence between knowledge production and disease control. Conversely, some regions exhibited inverse relationships, higher publication counts alongside increased estimated treatment needs for lymphatic filariasis or composite NTDs metrics, implying potential gaps between scientific focus and operational implementation.

Despite these inconsistencies, hierarchical models confirmed that, at the global level, increases in NTDs research were associated with modest yet statistically significant reductions in treatment requirements and anemia burden attributable to NTDs.

#### 3.2.5 Mortality indicators related to neglected diseases

Mortality patterns suggested further regional asymmetry. In South-East Asia, each additional publication was linked to declines in rabies, cysticercosis, and dengue deaths, whereas in Africa, significant associations were limited to snakebite mortality. These findings suggest that while some high-burden settings experience measurable mortality reductions aligned with research expansion, others may remain constrained by implementation or health-system capacity.

### 3.3 General health and system-level indicators

When broader health metrics were analyzed, coherence became more complex. Across most WHO regions, increased NTDs research activity correlated with higher absolute counts of deaths and DALYs, reflecting the concentration of research efforts in high-burden contexts rather than a causal worsening of indicators.

However, when mortality was standardized for population size, negative associations emerged: in Africa, each additional NTDs publication corresponded to an estimated reduction of nearly 200 deaths per standardized unit, and smaller yet significant decreases were observed in South-East Asia. These patterns suggest that, after adjusting for scale, research activity tends to accompany incremental improvements in population health.

In contrast, antibiotic consumption rates displayed consistent positive associations with NTDs research output across all regions. The strongest correlation occurred in the Eastern Mediterranean, likely reflecting concurrent expansion of diagnostic capacity, antimicrobial access, or surveillance systems accompanying scientific investment rather than direct causal effects.

Positive associations between research output and adverse health indicators reflect the concentration of scientific activity in high-burden contexts, rather than harmful effects of research itself.

### 3.4 Hierarchical mixed-effects models

**Figure 5:**
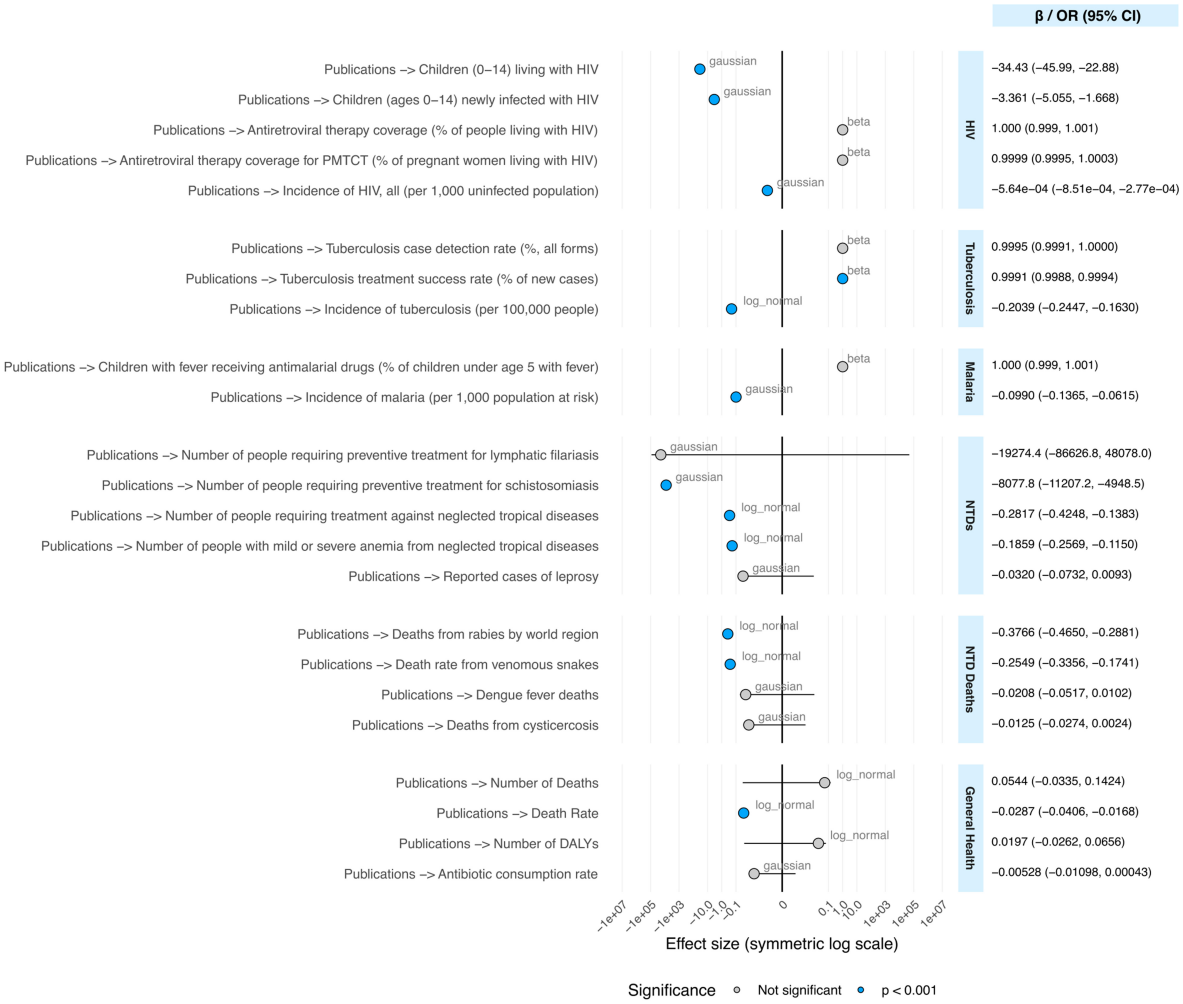
Hierarchical mixed-effects models of associations between neglected tropical diseases publications and health indicators. Forest plot displaying effect estimates from hierarchical mixed-effects models examining bidirectional relationships between neglected tropical diseases publication output and health indicators across 195 countries. Points represent regression coefficients with horizontal lines indicating 95% confidence intervals on a symmetric logarithmic scale. Arrow direction ( → ) indicates the predictor-outcome relationship being tested. Blue points indicate statistical significance at *p* <0.001 after Benjamini-Hochberg correction for multiple comparisons; gray points represent non-significant associations. Model distribution family is indicated next to each estimate (Gaussian, Gaussian_logit, Poisson, nbinom2, beta). All models included region level as a random effect to account for clustering and publication year as a covariate. Numerical values of effect estimates and confidence intervals are presented in the **right panel**. DALYs, disability-adjusted life years; NTDs, neglected tropical diseases; PMTCT, prevention of mother-to-child transmission.

When pooling data across all regions, mixed-effects models confirmed protective associations for HIV incidence (β = −0.0001; 95% CI: −0.0002 to −0.0000), malaria incidence (β = −0.09; 95% CI: −0.137 to −0.061), and schistosomiasis treatment need (β = −8,000; 95% CI: −11,200 to −4,900). Figure 5 summarizes the associations between NTD-related publication output and population-level indicators across WHO regions. Although Figure 5 reports a statistically significant overall association (*p* <0.001), effect sizes varied across regions, and statistical significance was not uniformly observed across all region-specific models.

In contrast, associations with DALYs and antibiotic consumption were non-significant after Benjamini–Hochberg adjustment (*p*-adj > 0.05).

### 3.5 Sensitivity analyses

Results remained robust across sensitivity checks. Excluding indicators with extensive missing data or re-estimating models using normalized publication ratios did not alter the direction or magnitude of coefficients. Reverse-specification models, where publication output was treated as the dependent variable, revealed weaker and often non-significant associations, suggesting that health improvements are unlikely to drive research activity alone.

## 4 Discussion

This study introduces a quantitative framework to evaluate how global research systems align with population health indicators, using NTDs as a model to explore what we call research coherence. By integrating bibliometric and epidemiological data across the six WHO regions over 25 years, we found that scientific productivity on NTDs has increased substantially and, in some domains, has coincided with measurable improvements in disease-specific indicators such as HIV, tuberculosis, and malaria. Yet, the alignment between research intensity and health progress remains partial, inconsistent, and unevenly distributed across regions and conditions.

These findings reflect both the success and the structural asymmetry of the research ecosystem related to NTDs (Bannach-Brown et al., 2025). Areas with strong translational infrastructure, sustained funding mechanisms, and established global partnerships (such as those historically developed for HIV and malaria) showed clearer coherence between scientific production and population-level indicators, consistent with prior evidence on differential research capacity and implementation readiness across diseases. In contrast, several NTDs are characterized by weaker implementation channels, understood here as limited access to late-phase clinical development, regulatory pathways, delivery platforms, and large-scale implementation mechanisms within health systems. These constraints are closely linked to structural inequities in the global research enterprise, including persistent imbalances in funding allocation, research capacity, and innovation ecosystems across diseases and regions, which in turn contribute to unequal health outcomes (Ioannidis, 2024). Importantly, observed misalignment between NTD-oriented research and burden indicators should be interpreted in light of the lengthy research-policy-impact loop and inherent time lags in knowledge translation, rather than as evidence of scientific failure. Instead, these patterns serve as a diagnostic signal of where the global research system is effectively aligned with population needs and where recalibration may be required (Ioannidis, 2024).

From a meta-epidemiological and meta-research perspective, coherence is not a binary measure of success but a dynamic indicator of alignment between knowledge generation, policy implementation, and societal benefit (Ioannidis, 2024; Lozada-Martinez et al., 2025a). Traditional bibliometric indicators can describe productivity (Fontecha et al., 2021; Tebano et al., 2024), but they cannot assess whether research meaningfully aligns with population health. Our framework proposes an empirical bridge between these two domains, showing that research systems can and should be evaluated by their alignment with reductions in disease burden, not merely by the quantity of papers produced (Kirby et al., 2025; Revolledo Caicedo et al., 2025; Romero-Freile et al., 2025). This approach aligns with current calls for evidence-based research governance, and it could inform funding agencies, governments, and universities seeking to assess the real-world impact of their investments (Lozada-Martinez et al., 2024).

To move from diagnosis to solution, we suggest three practical strategies. First, coherence indicators could be integrated into research evaluation frameworks, linking scientific output to epidemiological change within health priority areas (Kirby et al., 2025). This would encourage funders to reward alignment between research and disease burden rather than citation-based prestige (Jancovich et al., 2024). Second, coherence analysis could support research portfolio optimization by informing strategic investment decisions in areas where high research activity has not translated into commensurate health gains, signaling inefficiencies in translation and the need for reallocation, redesign of priorities, or strengthened implementation pathways, as well as identifying contexts where modest investment yields disproportionate benefits (Chowdhury et al., 2016). Third, embedding coherence metrics in the monitoring of the Sustainable Development Goals would allow global agencies to identify where the translation of knowledge into health improvement is weakest and to allocate resources accordingly (Samet and Hussein, 2024). Together, these strategies shift evaluation from productivity to purpose.

At the regional level, the strongest coherence signals were observed in Africa and South-East Asia, regions that have benefited from large-scale international collaborations and disease-control programs (World Health Organization, 2024). Yet these same regions continue to face barriers in authorship equity, data infrastructure, and institutional autonomy (Forbes et al., 2023). True research coherence, therefore, cannot exist without epistemic justice (Bannach-Brown et al., 2025). Global health progress depends not only on producing more science but on ensuring that the generation, interpretation, and application of evidence are equitably distributed (Adsul et al., 2024). Strengthening regional research capacity, fostering local leadership, and ensuring bidirectional partnerships are essential steps toward transforming coherence from a statistical observation into a structural reality (Chowdhury et al., 2016).

Methodologically, this study demonstrates how open data can be repurposed to evaluate the functioning of the research ecosystem itself (Bannach-Brown et al., 2025). The multi-level longitudinal design allowed us to detect both temporal and contextual patterns, providing an analytical model that can be replicated for other health domains (Revolledo Caicedo et al., 2025; Romero-Freile et al., 2025). Nevertheless, the results should be interpreted with caution. The ecological nature of the analysis precludes causal inference; unmeasured factors such as health system reforms or concurrent funding shifts could partly explain observed associations. Publication data are imperfect proxies for research activity, and differential data completeness across regions may introduce bias. Future work should incorporate alternative indicators, such as clinical guideline uptake, policy adoption, and patient-centered outcomes, to refine coherence metrics and extend their interpretability beyond epidemiological indicators.

Measuring scientific coherence is not an endpoint but a tool for continuous improvement (Ioannidis, 2018). By identifying where alignment between research and health indicators is weak, coherence analyses can inform targeted interventions, capacity-building, equitable collaborations, and priority reallocation (Chowdhury et al., 2016; Jancovich et al., 2024; Samet and Hussein, 2024), designed to close the gap between knowledge production and public health benefit (Chowdhury et al., 2016; Jancovich et al., 2024; Samet and Hussein, 2024). This approach transforms meta-research from critique into contribution, enabling a more reflexive and accountable science (Ioannidis, 2018).

This study has limitations that should be considered when interpreting the findings. First, regional bias reflects systematic heterogeneity across WHO regions in baseline levels and temporal trajectories of both scientific production and population-level indicators, driven by long-standing differences in research capacity, disease burden profiles, and data availability (Du et al., 2025). Although region-stratified and mixed-effects models were used to mitigate this heterogeneity, residual differences may persist. Second, temporal dependency is inherent to the longitudinal structure of the data, as country-level indicators and publication counts are observed repeatedly over time and exhibit persistence and trend-related correlation across years (Du et al., 2025). While the selected modeling approach accounts for repeated observations and regional variation, it does not fully disentangle complex dynamic or delayed relationships. Accordingly, our findings should be interpreted as estimates of system-level scientific coherence rather than causal effects.

Despite longstanding interest in understanding whether scientific activity translates into measurable population-level health improvements, robust causal models linking research production to health indicators remain scarce (Du et al., 2025). This is not due to a lack of methodological development in clinical epidemiology or meta-research, but rather to structural constraints that have persisted over decades. These include limited linkage between bibliometric data, research funding, policy adoption, and health indicators; long, heterogeneous, and non-linear translational pathways from knowledge generation to implementation; regulatory and geopolitical variability across regions; and the ecological nature of most globally comparable health indicators (Sipido and Nagyova, 2020). As a result, causal inference approaches (while theoretically appealing) are often infeasible or weakly identified when applied to aggregated, region-year global data.

The limited strength of reverse associations indicates that research activity does not simply mirror epidemiological fluctuations, reinforcing the view of scientific systems as partially autonomous and influenced by structural and institutional determinants beyond disease burden alone.

Within this context, the present study does not aim to estimate causal effects. Instead, we propose and operationalize a longitudinal, ecological proxy of what we term scientific coherence. By examining temporal co-variation rather than causal attribution, this approach provides a pragmatic and reproducible framework to assess whether research activity responds to health needs at the system level. We position this framework as hypothesis-generating and complementary to, rather than a substitute for, future causal or quasi-causal studies that may become feasible as data integration and methodological infrastructures advance.

An additional consideration is the potential time lag between scientific production and measurable changes in population-level health indicators (Du et al., 2025; Wang et al., 2021). While such lags are central to causal interpretations of research impact, the present study focuses on estimating temporal alignment rather than delayed effects. Our models therefore capture contemporaneous co-variation between publication volume and health indicators, reflecting whether scientific activity evolves in synchrony with population health needs (Wang et al., 2021). We acknowledge that delayed effects may exist and that future work could explore lagged structures as data integration improves; however, the absence of an explicit lag does not invalidate the assessment of scientific coherence as defined here.

The rationale for examining the alignment between NTD-focused scientific production and broader population-level health indicators is grounded in an exploratory, systems-oriented perspective. From the standpoint of the social determinants of health, scientific research and its translation are not disease-isolated processes, but human-dependent and dynamic social activities embedded within health systems, policies, and institutional capacities. Consequently, research agendas oriented toward NTDs may reflect (and potentially accompany) broader systemic changes that influence multiple health domains beyond disease-specific indicators.

This study does not claim that NTD-specific research causally improves outcomes related to other major infectious diseases. Rather, it explores whether the evolution of NTD-oriented scientific production exhibits temporal alignment with a wider set of population health indicators, consistent with a system-level coherence framework. We acknowledge that spillover effects from research on major infectious diseases toward NTD outcomes represent an alternative and plausible direction, which is supported by existing literature. Exploring such reverse or bidirectional dynamics constitutes an important avenue for future research. Our approach is therefore positioned as exploratory and hypothesis-generating, aimed at capturing complex science-society interactions rather than asserting directional impact.

As highlighted in recent meta-research, a substantial proportion of health research remains preclinical, and the translation from knowledge generation to measurable patient or population-level benefits often spans many years or decades. This lengthy research-to-evidence pathway further constrains the feasibility of attributing short-term or contemporaneous changes in population health indicators to scientific output. Accordingly, the associations observed in this study should be interpreted within the context of delayed and indirect translation processes, reinforcing the non-causal and system-level nature of the scientific coherence framework.

More broadly, this work aligns with emerging frameworks of responsible research evaluation and evidence-informed research governance, which advocate moving beyond productivity-based metrics toward assessments that reflect societal relevance, health needs, and system-level outcomes.

## 5 Conclusion

These findings highlight that while research on NTDs has supported progress in several key areas, the scientific coherence between scientific productivity and population health remains fragmented. Addressing this fragmentation requires not only describing incoherence but actively designing mechanisms to correct it. The proposed framework and coherence indicators offer a practical means to do so, redefining research evaluation as a tool for alignment, accountability, and equity in the global evidence ecosystem.

## Data Availability

The original contributions presented in the study are included in the article/Supplementary material, further inquiries can be directed to the corresponding authors.
