# Supplementary material for: Measuring scientific coherence between global neglected tropical disease research and population health indicators: a 25-year meta-research study
Source: Front Res Metr Anal. 2026 Jun 18;11:1766718. doi: 10.3389/frma.2026.1766718 (PMC13323324; doi:10.3389/frma.2026.1766718)
Supplement: Supplementary file 1 [file Table_1.docx]

**Supplementary Material #1**

Search strategies used:

***Scopus database***

TITLE("Neglected Diseases") OR TITLE("Neglected Disease") OR TITLE("Neglected Tropical Diseases") OR TITLE("Neglected Tropical Disease") OR TITLE("Lymphatic Filariasis") OR TITLE("Filarial Elephantiases") OR TITLE("Filarial Elephantiasis") OR TITLE("Lymphatic Filariases") OR TITLE("Lymphatic Filariasis") OR TITLE("Bancroftian Elephantiases") OR TITLE("Wuchereria bancrofti Infection") OR TITLE("Bancroftian Filariasis") OR TITLE("Bancroftian Filariases") OR TITLE("Bancroftian Elephantiasis") OR TITLE("Malayi Filariasis") OR TITLE("Malayi Filariases") OR TITLE("Brugian Filariasis") OR TITLE("Brugian Filariases") OR TITLE("Malayi Elephantiases") OR TITLE("Malayi Elephantiasis") OR TITLE(Onchocerciasis) OR TITLE(Schistosomiasis) OR TITLE(Schistosomiases) OR TITLE("Schistoma Infection") OR TITLE("Schistoma Infections") OR TITLE(Bilharziasis) OR TITLE(Bilharziases) OR TITLE("Katayama Fever") OR TITLE(Helminthiasis) OR TITLE(Helminthiases) OR TITLE("Nematomorpha Infection") OR TITLE("Nematomorpha Infections") OR TITLE(Trachoma) OR TITLE("Egyptian Ophthalmia") OR TITLE(Leprosy) OR TITLE("Hansen Disease") OR TITLE("Hansen's Disease") OR TITLE(Dracunculiasis) OR TITLE(Dracunculosis) OR TITLE(Dracunculoses) OR TITLE("Guinea Worm Disease") OR TITLE("Guinea Worm Diseases") OR TITLE("Guinea Worm Infection") OR TITLE(Yaws) OR TITLE("Frambesia Tropica") OR TITLE("Frambesia Tropicas") OR TITLE(Frambesia) OR TITLE(Frambesias) OR TITLE("Human African Trypanosomiasis") OR TITLE("Chagas Disease") OR TITLE("Trypanosoma cruzi Infection") OR TITLE("Trypanosoma cruzi Infections") OR TITLE("Chagas' Disease") OR TITLE("South American Trypanosomiasis") OR TITLE("American Trypanosomiasis") OR TITLE("Visceral Leishmaniasis") OR TITLE("Kala-Azar") OR TITLE("Kala Azar") OR TITLE("Black Fever") OR TITLE(Rabies) OR TITLE("Encephalitic Rabies") OR TITLE("Soil-Transmitted Helminthiases") OR TITLE("Buruli Ulcer") OR TITLE("Mycobacterium ulcerans Infections") OR TITLE("Mycobacterium ulcerans Infection") OR TITLE(Dengue) OR TITLE("Break-Bone Fever") OR TITLE("Break Bone Fever") OR TITLE("Breakbone Fever") OR TITLE(Chikungunya) OR TITLE("Chikungunya Fever") OR TITLE("Chikungunya Virus Infection") OR TITLE("Chikungunya Virus Infections") OR TITLE(Echinococcosis) OR TITLE("Echinococcus Infection") OR TITLE("Echinococcus Infections") OR TITLE("Echinococcus granulosus Infection") OR TITLE("Echinococcus granulosus Infections") OR TITLE("Foodborne Trematodiases") OR TITLE("Cutaneous Leishmaniasis") OR TITLE("Cutaneous Leishmaniases") OR TITLE("American Leishmaniasis") OR TITLE("Oriental Sore") OR TITLE(Mycetoma) OR TITLE(Maduromycosis) OR TITLE(Eumycetoma) OR TITLE(Actinomycetoma) OR TITLE(Chromoblastomycosis) OR TITLE(Chromomycosis) OR TITLE(Chromomycoses) OR TITLE(Scabies) OR TITLE("Sarcoptic Mange") OR TITLE("Snakebite Envenoming") OR TITLE("Snake Bite") OR TITLE(Snakebites) OR TITLE(Snakebite) OR TITLE("Snake Envenomings") OR TITLE("Snakebite Envenomation") OR TITLE("Snakebite Envenomations") OR TITLE("Snake Envenomations") OR TITLE(Taeniasis) OR TITLE(Taeniases) OR TITLE("Taenia Infection") OR TITLE("Taenia Infections") OR TITLE("Taenia glomeratus Infection") OR TITLE("Taenia solium Infection") OR TITLE("Taenia solium Infections") OR TITLE("Taenia serialis Infections") OR TITLE("Taenia multiceps Infection") OR TITLE("Taenia brauni Infection") OR TITLE(Cysticercosis) OR TITLE(Cysticercoses) OR TITLE(Coenurosis) OR TITLE(Coenuroses) OR TITLE("Coenuri Infection") OR TITLE("Coenuri Infections") OR TITLE("Coenurus Infection") OR TITLE("Coenurus Infections") OR TITLE("Coenurus cerebralis Infection") OR TITLE("Coenurus cerebralis Infections") OR TITLE("Cysticercus cellulosae Infection") OR TITLE("Cysticercus cellulosae Infections") OR TITLE("Taenia solium Cysticercosis") OR TITLE("Taenia solium Cysticercoses") = 143,475 documents found.
