# Supplementary material for: Measuring scientific coherence between global neglected tropical disease research and population health indicators: a 25-year meta-research study
Source: Front Res Metr Anal. 2026 Jun 18;11:1766718. doi: 10.3389/frma.2026.1766718 (PMC13323324; doi:10.3389/frma.2026.1766718)
Supplement: Supplementary file 3 [file Table_3.docx]

Supplementary Material

**Supplementary Table 1. Indicators extracted for the analyses (N = 75).**

| **Category** | **Indicator** | **Variable Type** |
| --- | --- | --- |
| **Health System and Healthcare Access** | Current Health Expenditure (% of GDP) | Independent |
|  | Healthcare Access and Quality | Dependent |
|  | Nurses and Midwives (per 1,000 people) | Independent |
|  | Out-of-Pocket Expenditure on Health | Independent |
|  | Physicians (per 1,000 people) | Independent |
| **Disease Burden and Infectious Diseases** | Antibiotic consumption rate | Dependent |
|  | Antiretroviral therapy coverage (% of people living with HIV) | Dependent |
|  | Antiretroviral therapy coverage for PMTCT (% of pregnant women living with HIV) | Dependent |
|  | Children (0-14) living with HIV | Dependent |
|  | Children (ages 0-14) newly infected with HIV | Dependent |
|  | Children with fever receiving antimalarial drugs (% of children under age 5 with fever) | Dependent |
|  | Death Rate | Dependent |
|  | Death rate from venomous snakes | Dependent |
|  | Deaths from cysticercosis | Dependent |
|  | Deaths from rabies by world region | Dependent |
|  | Dengue fever deaths | Dependent |
|  | Incidence of HIV, all (per 1,000 uninfected population) | Dependent |
|  | Incidence of malaria (per 1,000 population at risk) | Dependent |
|  | Incidence of tuberculosis (per 100,000 people) | Dependent |
|  | Number of DALYs | Dependent |
|  | Number of Deaths | Dependent |
|  | Number of people requiring preventive treatment for lymphatic filariasis | Dependent |
|  | Number of people requiring preventive treatment for schistosomiasis | Dependent |
|  | Number of people requiring treatment against neglected tropical diseases | Dependent |
|  | Number of people with mild or severe anemia from neglected tropical diseases | Dependent |
|  | Reported cases of leprosy | Dependent |
|  | Tuberculosis case detection rate (%, all forms) | Dependent |
|  | Tuberculosis treatment success rate (% of new cases) | Dependent |
| **Demographic and Population Health** | Population, ages 65+ | Independent |
|  | Child mortality rate | Dependent |
|  | Life expectancy at birth | Dependent |
|  | Sex ratio | Independent |
|  | Sex gap in life expectancy | Independent |
|  | Healthy life expectancy | Dependent |
|  | Lifespan Inequality in women | Independent |
|  | Lifespan Inequality in men | Independent |
|  | International migrant stock, total | Independent |
|  | Prevalence of anemia among pregnant women (%) | Dependent |
|  | Homelessness rate | Dependent |
|  | Population in year | Independent |
| **Economic and Poverty** | Gini Coefficient | Independent |
|  | Multidimensional Poverty Index | Independent |
|  | Number of people living in extreme poverty | Independent |
|  | Income inequality: Atkinson index | Independent |
|  | Prevalence of moderate or severe food insecurity in the population (%) | Dependent |
|  | Prevalence of severe food insecurity in the population (%) | Dependent |
|  | GDP per capita | Independent |
| **Education and Development** | Average years of schooling | Independent |
|  | Elderly Literacy Rate | Independent |
|  | Youth Literacy Rate | Independent |
|  | Human Development Index | Independent |
|  | Share of population with no formal education | Independent |
| **Governance and Political** | Private civil liberties index | Independent |
|  | LGBT+ legal equality index | Independent |
|  | Percentage of territory effectively controlled by government | Independent |
|  | Rigorous and impartial public administration index | Independent |
|  | State capacity index | Independent |
|  | Functioning government index | Independent |
|  | Political corruption index | Independent |
|  | Corruption Perception Index | Independent |
|  | Human rights index | Independent |
| **Water, Sanitation, and Hygiene** | Share of deaths attributed to unsafe sanitation | Dependent |
|  | Share of people practicing open defecation | Dependent |
|  | Share of the population using basic sanitation service | Dependent |
|  | Share of the population using safely managed drinking water sources | Dependent |
|  | Share of the population with access to basic handwashing facilities | Dependent |
| **Research and Development** | Annual research & development funding for infectious diseases | Independent |
|  | Charges for the use of intellectual property, payments (BOP, current US$) | Independent |
|  | Distribution of R&D funding flows for neglected diseases by country | Independent |
|  | Drugs pipeline for neglected tropical diseases by country | Dependent |
|  | Drugs pipeline for neglected tropical diseases by income | Dependent |
|  | Drugs pipeline for neglected tropical diseases by region | Dependent |
|  | Higher education institutions offering disciplines related to research for health in 2023 by region | Independent |
|  | Higher education institutions offering disciplines related to research for health in 2023 by income | Independent |
|  | Research and Development Expenditure (% of GDP) | Independent |
